# Simple Reason for Hypoglycemia: ACE Inhibitor-induced Severe Recurrent Hypoglycemia in a Nondiabetic Patient

**DOI:** 10.7759/cureus.5449

**Published:** 2019-08-21

**Authors:** Ghada Elshimy, Pawarid Techathaveewat, Mahmoud Alsayed, Sathya Jyothinagaram, Ricardo Correa

**Affiliations:** 1 Endocrinology, Diabetes and Metabolism, University of Arizona College of Medicine-Phoenix, Phoenix, USA

**Keywords:** lisinopril, ace inhibitors, hypoglycemia, hypertension

## Abstract

Angiotensin-converting enzyme (ACE) inhibitors are among the most common medications used to treat patients with concomitant diabetes and hypertension. They are considered the first line of treatment for hypertension in this population. Several case studies have reported that ACE inhibitors can induce hypoglycemia in patients with diabetes. To our knowledge, however, ACE inhibitors have not been found to induce hypoglycemia in patients without diabetes. This report describes a patient without diabetes experiencing recurrent severe hypoglycemia induced by the ACE inhibitor lisinopril.

## Introduction

Angiotensin-converting enzyme (ACE) inhibitors are among the most common medications used to treat patients with concomitant hypertension and diabetes. They are considered the first line of treatment for hypertension in this population. ACE inhibitors can improve insulin sensitivity, which can lead to an approximately three- to four-fold increase in the risk of hypoglycemia in patients with diabetes, especially those receiving other hypoglycemic agents, in particular, sulfonylureas. In contrast, treatment with angiotensin receptor blockers is not associated with an increased risk of hypoglycemia. To our knowledge, ACE inhibitors have not been reported to induce hypoglycemia in patients without diabetes [1-7]. This report describes a patient without diabetes experiencing recurrent severe hypoglycemia induced by the ACE inhibitor lisinopril.

## Case presentation

**Figure 1:**
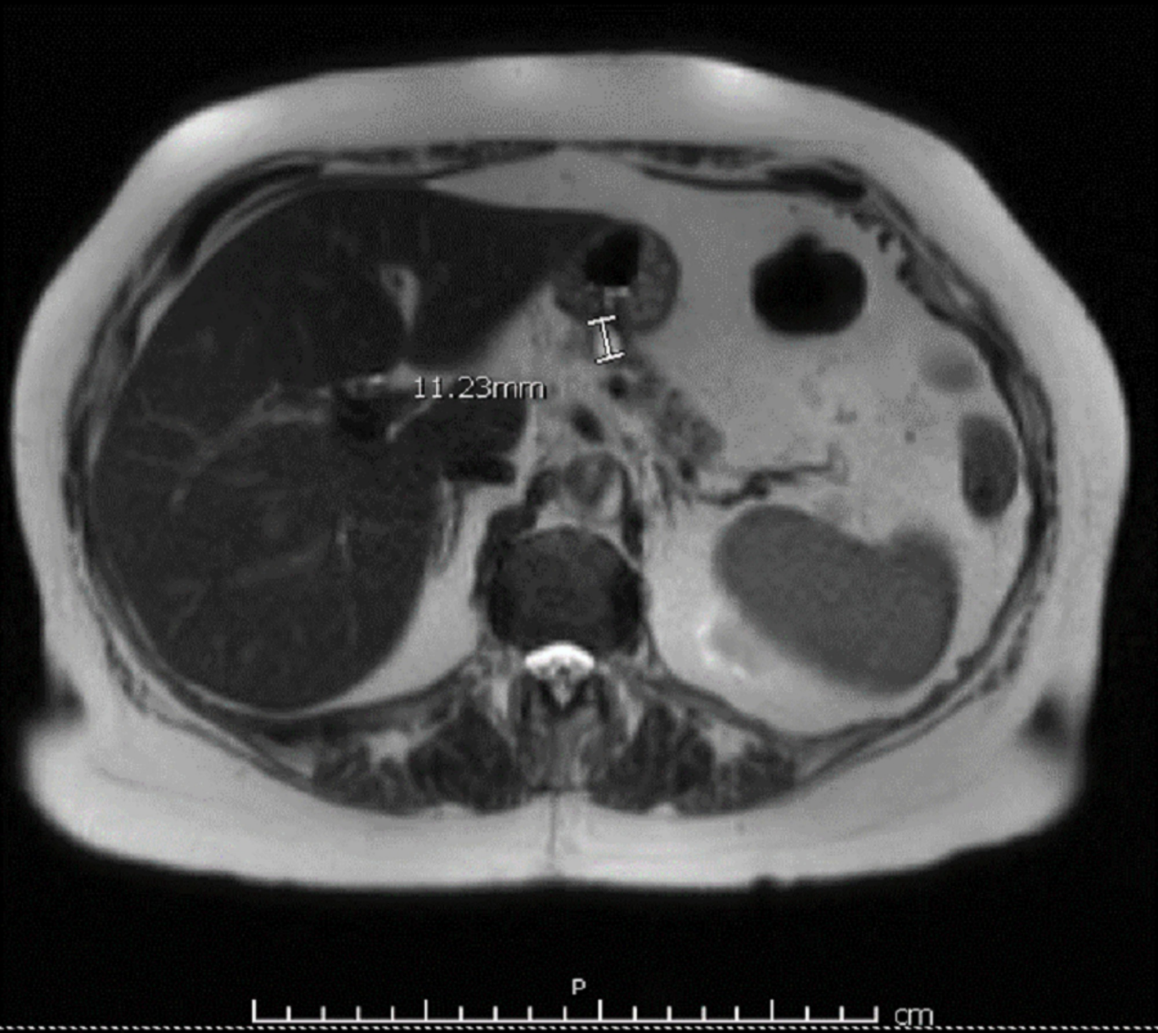
Abdominal MRI with and without contrast showing a 1.1-cm cystic lesion in the body of the pancreas without internal enhancement or septations.

A 76-year-old woman with a past medical history of hypertension and no prior history of diabetes who was being treated with lisinopril presented to the ED with an abrupt onset of dysarthria and confusion. She was found to have hypoglycemia (blood glucose, 25 mg/dL), which responded to intravenous administration of 50 mL of 50% dextrose, with complete resolution of her symptoms. However, hypoglycemia recurred in less than one hour, and she was started on a 10% dextrose drip. Abdominal MRI showed a cystic pancreatic lesion around 1.1 cm in diameter, which was confirmed by endoscopic ultrasound (Figure 1).

 The results of all other tests were within normal ranges, including high dose cosyntropin (250 µg) stimulation test, hemoglobin A1C concentration, and thyroid, kidney, and liver function tests (Table 1).

**Table 1:** Normal results of cosyntropin (synthetic ACTH) stimulation test, following intravenous injection of a standard dose (250 µg) at baseline. ACTH, adrenocorticotropic hormone.

Cortisol level at baseline	11.4 µg/dL
Cortisol level 30 min after receiving cosyntropin	21.9 µg/dL
Cortisol level 60 min after receiving cosyntropin	25.0 µg/dL

The patient was discharged but was readmitted a few days later with recurrent hypoglycemia. A 72-h fasting glucose test showed noninsulin-mediated hypoglycemia, with a blood glucose concentration of 51 mg/dL that occurred only after 10 h of fasting. Other tests showed an insulin concentration of 2 IU/mL, a c-peptide concentration of 0.7 ng/mL, a proinsulin concentration <7.5 pmol/L, an anti-insulin antibody concentration <0.4 uU/mL, negative results on a urine sulfonylurea screening test, and an elevated beta-hydroxybutyrate concentration of 23.4 mmol/L (Table 2).

**Table 2:** Serum concentrations of various factors following a 72-h fasting test performed during the patient’s second hospitalization.

Factor	Concentration
Blood glucose	51 mg/dL
Beta-hydroxybutyrate	23.4 mg/dL (normal, ≤2.8 mg/dL)
C-peptide	0.7 ng/mL (normal, 1.1–4.4 ng/mL)
Proinsulin	<7.5 pmol/L (normal, ≤18.8 pmol/L)
Insulin	2 uIU/mL (normal, 2–25 µIU/mL)
Insulin Ab	< 0.4 U/mL (normal, <0.4 U/mL)
Human growth hormone	0.8 ng/mL (normal, ≤7.9 ng/mL)

**Figure 2:**
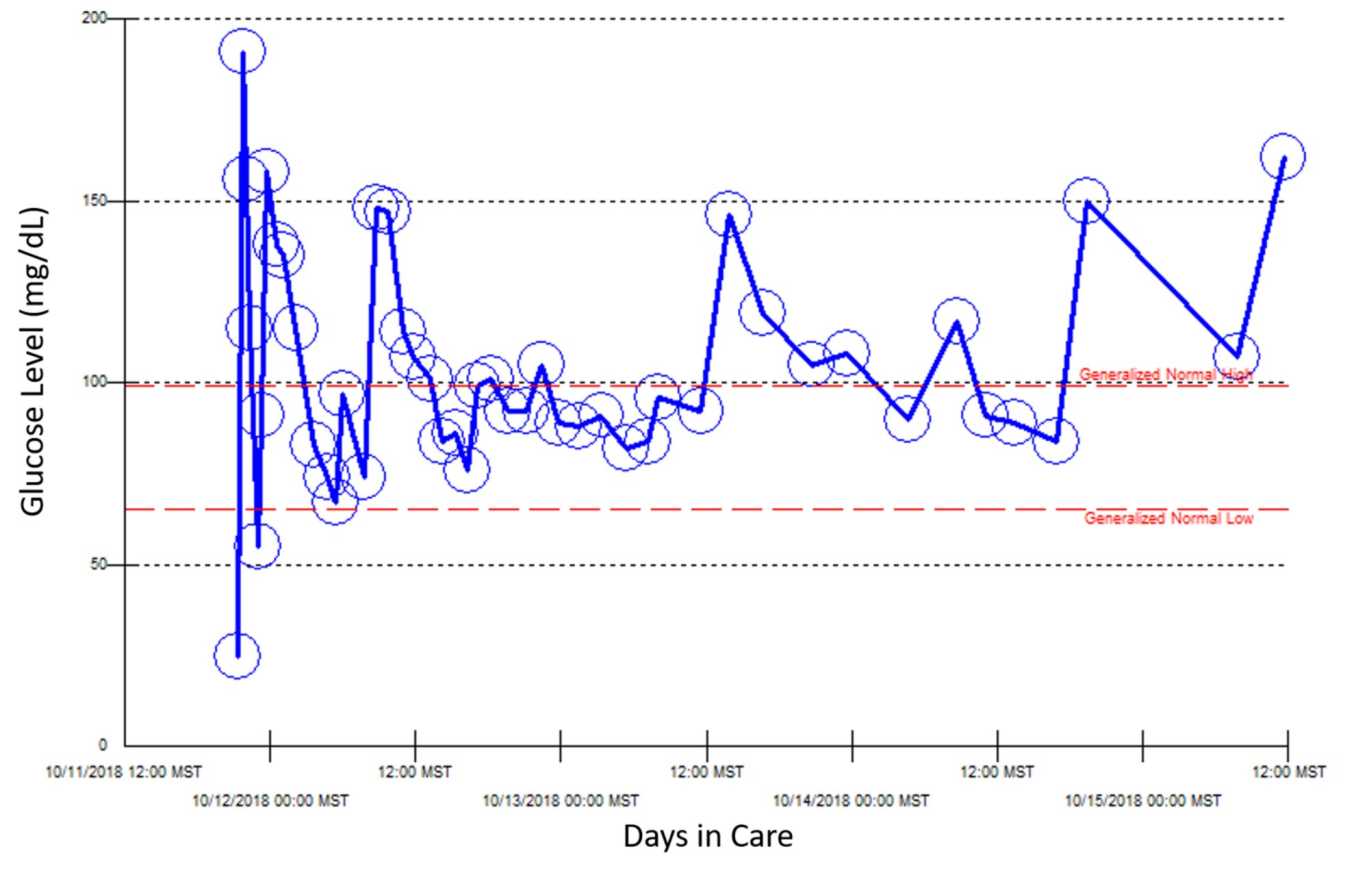
Point of care blood glucose reading during the patient’s first hospital admission, October 11–15, 2018.

**Figure 3:**
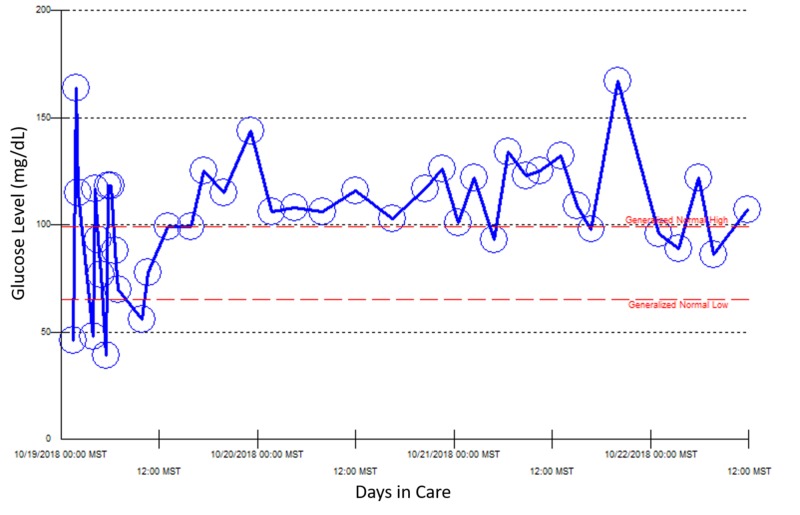
Point of care blood glucose reading during the patient’s second hospital admission, October 19–22, 2018.

**Figure 4:**
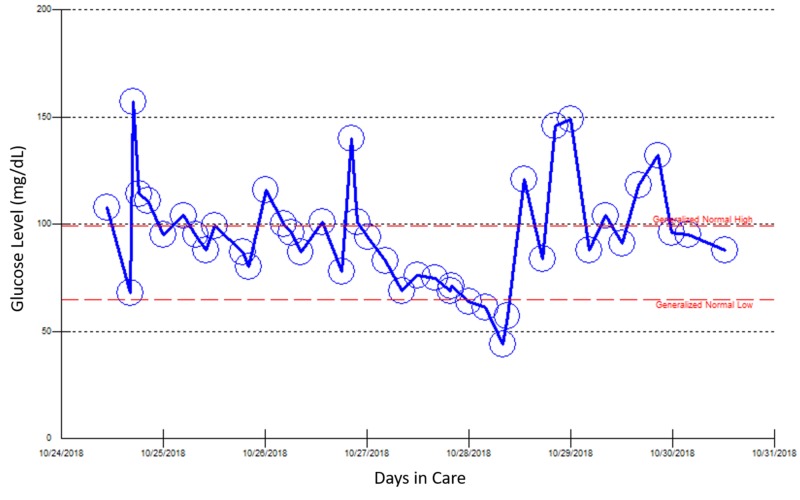
Point of care blood glucose reading during the patient’s third hospital admission, October 24–30, 2018. At this admission, the patient presented with left leg swelling and deep vein thrombosis secondary to diazoxide treatment for two days.

**Figure 5:**
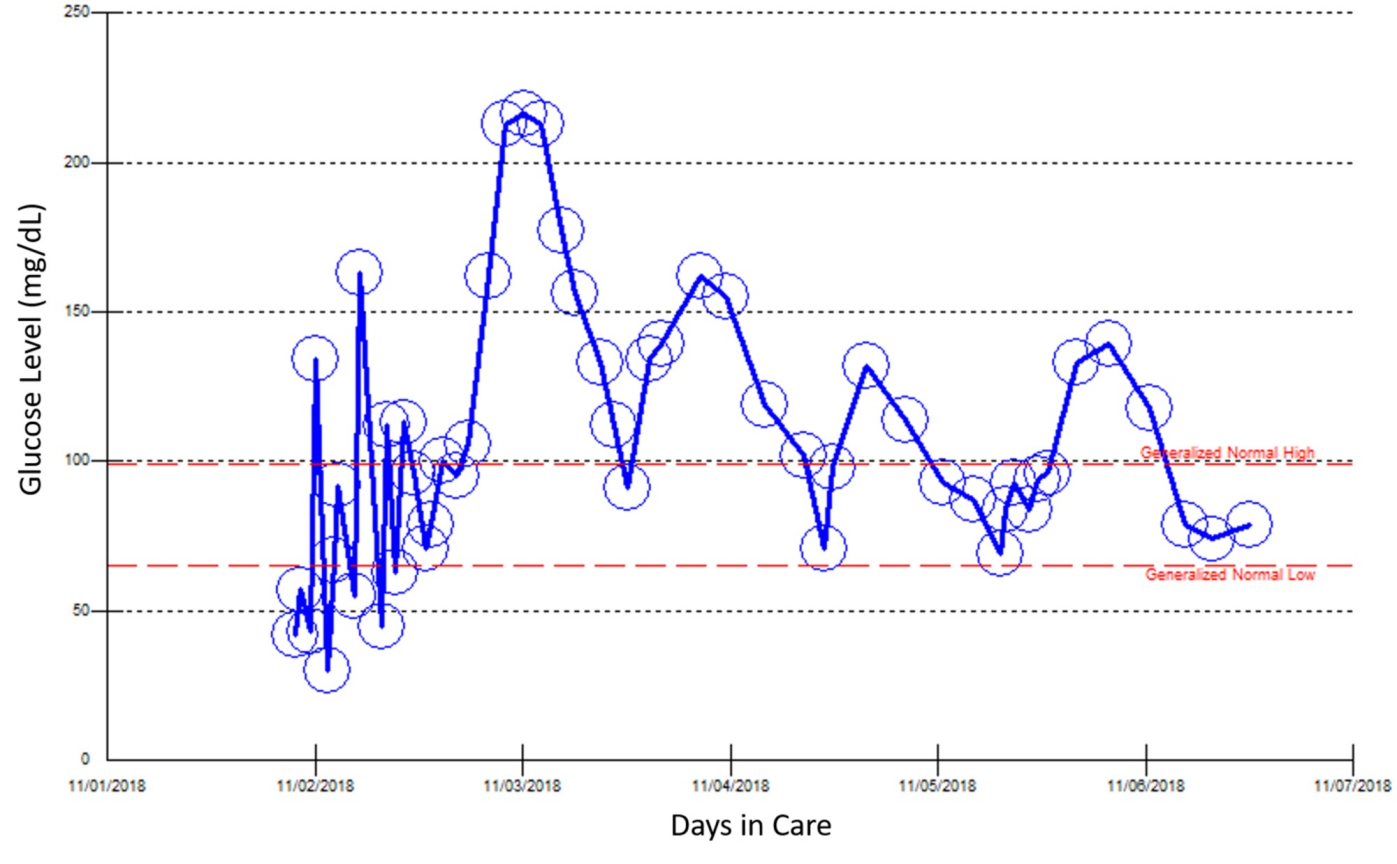
Point of care blood glucose reading during the patient’s fourth hospital admission, November 1–6, 2018.

Because she required a continuous 10% dextrose drip for a few days, the patient was started on diazoxide to control her blood glucose concentration. However, she developed deep venous thrombosis three days later, which led to discontinuation of diazoxide. Because she was admitted multiple times for recurrent hypoglycemia over three weeks, other causes of hypoglycemia were investigated (Figures 2-5).

A review showed that these episodes of hypoglycemia only occurred two weeks after her dose of lisinopril was increased from 10 mg to 20 mg for her uncontrolled hypertension. Lisinopril was discontinued, and the patient started on treatment with losartan. Monitoring of her blood glucose concentration showed that no additional episodes of hypoglycemia occurred after discontinuation of lisinopril.

## Discussion

Hypoglycemia is defined as a serum blood glucose level concentration <70 mg/dL accompanied by the Whipple triad. Symptoms of hypoglycemia may be mild (adrenergic symptoms) or worrisome (neuroglycopenic symptoms, including seizures), with the intensity of symptoms often being directly related to the level of hypoglycemia. The etiology of hypoglycemic events in patients with diabetes is usually related to the treatment of diabetes, such as overly high doses of insulin or a sulfonylurea. These episodes are considered nonalarming as patients can be treated quickly. The etiology of recurrent hypoglycemia in a patient without diabetes, however, is unclear, often requiring extensive patient workup. Causes of hypoglycemia in patients without diabetes may be as simple as adrenal insufficiency or as rare as endocrine neoplasia such as insulinoma [6, 8-10].

The ACE inhibitors are antihypertensive agents commonly used to lower blood pressure in both patients with and without diabetes. These agents reduce angiotensin II levels, resulting in blood pressure reductions due to the relaxation of efferent vessels of the glomeruli, lowering glomerular pressure and decreasing proteinuria, thereby reducing the likelihood of diabetic nephropathy. The ACE inhibitors also alter expression of the hormone bradykinin, which has been shown to have a tussive-effect through the kininogen-kinin system, leading to cough as a side effect, an etiology associated with hypoglycemia [6-10].

The ACE inhibitor captopril was first reported in 1985 to induce hypoglycemia in four hypertensive patients, three with insulin-dependent diabetes and one with noninsulin dependent diabetes [5]. Although the exact mechanism by which ACE inhibitors induce hypoglycemia remains unclear, these agents are thought to indirectly increase insulin sensitivity by increasing the levels of circulating kinins which, in turn, leads to increased glucose uptake by muscle tissue through a vasodilatation effect and also downregulate hepatic glucose production [3, 5]. A case report in 1986 described two patients with diabetes who required reductions in their dose of insulin or hypoglycemic agents after treatment with enalapril for hypertension [6]. Captopril treatment of two additional patients with noninsulin-dependent diabetes resulted in hypoglycemia [7]. Experimental studies suggested that muscle response to insulin is increased in rats with streptozotocin-induced diabetes and in patients with noninsulin-dependent diabetes, suggesting that ACE inhibitors reduce glucose concentrations by elevating systemic kinin levels [9]. In 1997, a patient with diabetes experienced alacepril-induced hypoglycemia complicated by nephropathy, with hypoglycemia in this patient correlating with the use of ACE inhibitors and impaired kidney function [11].

The binding of bradykinin to bradykinin type 2 receptors may have a direct effect on insulin-mediated glucose absorption in the body. Bradykinin-induced reductions in angiotensin II concentrations may also blunt sympatho-adrenergic responses to insulin [3, 12-13]. To date, the exact mechanism by which ACE inhibitors induce hypoglycemia remains unclear.

Our patient underwent extensive testing to rule out all other possible causes of hypoglycemia, such as adrenal insufficiency and IGF-2 producing tumors, with insulin-mediated processes ruled out by 72-h fasting. Hypoglycemia in this patient disappeared after discontinuation of the ACE inhibitor, confirming the diagnosis of lisinopril-induced severe hypoglycemia. Hypoglycemia in this patient was likely due to increased insulin sensitivity, but the exact mechanism remains incompletely understood, emphasizing the need for further investigations in animal models.

## Conclusions

Hypoglycemia is a very rare side effect of ACE inhibitors in patients without diabetes. The results of the present study indicate that patients without diabetes may experience ACE inhibitor-induced hypoglycemia, which responds to discontinuation of the medication as in our patient. Patient medications should be reviewed periodically when managing hypoglycemia.
